# Liver Fibrosis and Inflammation under the Control of ERK2

**DOI:** 10.3390/ijms21113796

**Published:** 2020-05-27

**Authors:** Kuo-Shyang Jeng, Ssu-Jung Lu, Chih-Hsuan Wang, Chiung-Fang Chang

**Affiliations:** Division of General Surgery and Department of Medical Research, Far Eastern Memorial Hospital, No.21, Sec. 2, Nanya S. Rd., Banciao Dist., New Taipei City 220, Taiwan; kevin.ksjeng@gmail.com (K.-S.J.); sunnylu312@gmail.com (S.-J.L.); joyce.walawala@gmail.com (C.-H.W.)

**Keywords:** ERK2, MAPK, liver cirrhosis, liver inflammation

## Abstract

Chronic liver injury could lead the formation of liver fibrosis, eventually some would develop to hepatocellular carcinoma (HCC), one of the leading malignancies worldwide. The aim of the study is to dissect the role of extracellular signal-regulated kinase 2 (ERK2) signaling in liver fibrosis and inflammation. The choline-deficient, ethionine-supplemented (CDE) diet could lead to fatty livers and generate oval cells, activate hepatocyte stellate cell (HSC) and recruit immune cells as the liver fibrosis model mice. WT and ERK2 deficient (ERK2^−/−^) mice were compared in terms of liver weight/body weight, liver function, liver fibrosis markers and the differential gene expression in hepatotoxicity. ERK2^−/−^ mice display the less degree of liver fibrosis when compared to WT mice. The protein level of alpha smooth muscle (α-SMA) was reduced and several hepatocellular carcinoma-related genes such as MMP9, FoxM1 were down-regulated. In addition, the cell proliferation and the percentages of activated T cells were reduced in ERK2^−/−^ mice upon liver injury. Therefore, ERK2 plays an important role in regulating liver cirrhosis and inflammation.

## 1. Introduction

Liver fibrosis is one of major causes of morbidity worldwide [1,2]. Aberrant apoptotic hepatocytes and activated hepatic stellate cells (HSC) are involved in the process of liver cirrhosis. Alpha smooth muscle (alpha-SMA) is a marker for liver fibrosis [3,4]. In chronic liver diseases, liver cell death and collagen accumulation could lead to release chemokines for recruiting immune cells [5,6]. Long-term irreversible liver damage could result in advanced liver diseases such as hepatocellular carcinoma (HCC), one of the leading causes of cancer-related deaths worldwide [7,8]. The treatment of HCC is still a challenge, with 1-year survival rates of 20% and a median survival of 8 months [9]. Sorafenib is a multi-kinase inhibitor as the first line treatment for the late stage HCC [10,11]. However, it could only extend the median survival by about three months. Therefore, it is important to understand the detail mechanism of liver cirrhosis for developing potential diagnosis biomarkers or drug target in preventing its progression to HCC [12].

Extracellular signal-regulated kinase (ERK) belongs to mitogen-activated protein kinase (MAPK) pathway, which the cascade is under the control of RAF protein, MEK1/2 and ERK1/2. The phosphorylation of ERK could directly regulate the downstream gene expression. Erk1 deficient mice display normal liver function whereas ERK2 plays a pivotal mediator in hepatocyte cell cycle [13,14]. ERK1 deficiency mice display normal protein level of the cell cycle related protein and kinase for proliferation [13]. Increased ERK activity contributes to liver energy metabolism [15]. Besides, ERK2 could be involved in the suppression of ER stress in high sucrose and high fat diet [16]. The down-regulation of RAF kinase inhibitory protein (RKIP) promotes ERK signaling and thereby results in serious liver fibrosis [17].

The simultaneous activation of ERK and AKT pathways enhance cell cycle progression in Hepatitis B virus (HBV)-replicating hepatocytes whereas HCV envelop protein activates the ERK pathway to facilitate human hepatoma cell proliferation and survival [18]. ERK signaling pathway plays a crucial role in HCC cell growth, cell migration and epithelial-mesenchymal transition (EMT) development [19,20,21]. The phosphorylation of ERK could be a potential marker to represent the sensitivity of sorafenib for HCC treatment and show a significant correlation with a decreased overall survival [22,23]. ERK substrate Egr1 has been found to promote angiogenesis, fibrogenesis and tumorigenesis in HCC [24]. Inhibition of MAPK signaling pathway could reduce cell proliferation, decrease cancer stem cell expression and increase apoptosis in hepatoma cells [25,26,27].

Patients with liver fibrosis/cirrhosis have higher risks of developing HCC [7,28]. By understanding the detail mechanism for ERK signaling in liver fibrosis would be helpful to identify potential targets to prevent the process from liver fibrosis to HCC. In this study, the role of ERK2 in liver fibrosis and liver inflammation would be investigated in transgenic mice (Erk2 deficient mice) under the liver fibrosis mouse model.

## 2. Results

### 2.1. Erk2-Deficient Mice Displayed Normal Liver but Reduced Body Weight under the Normal Chow Diet

**Figure 1:**
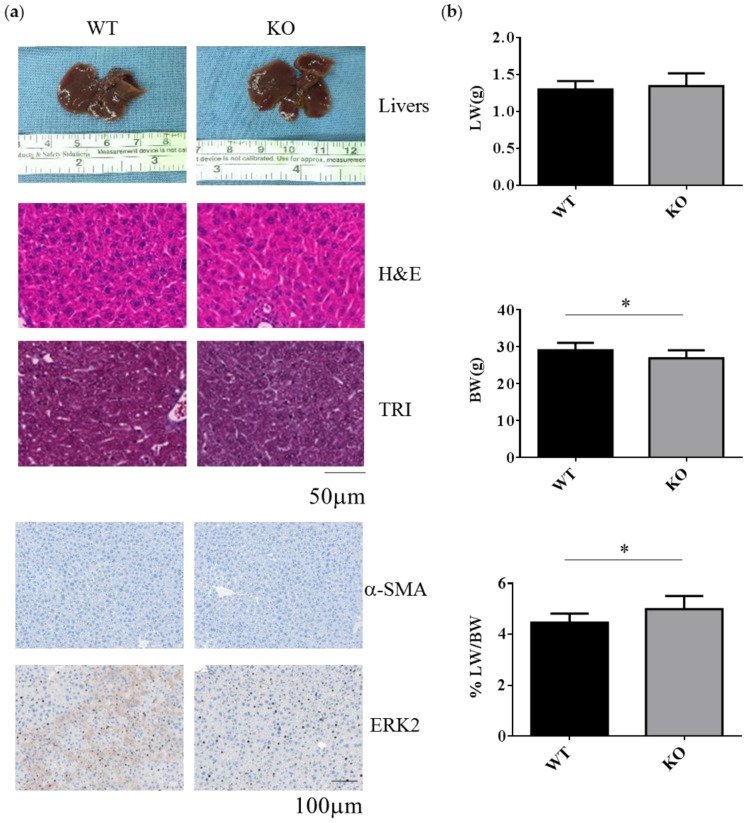
Decreased body weight and increased the percentages of liver weight to body weight in Erk2^−/−^ (KO) livers. (**a**) The representative livers, liver histology (H&E, hematotoxylin and eosin staining), liver fibrosis (TRI, Trichrome staining) or immunostaining of α-SMA and ERK2 of the WT and Erk2^−/−^ (KO) mice was under the chow diet (normal diet) treatment; (**b**) The liver weights (LW), body weight (BW), and the percentages of liver weight to body weight in Wild-type (WT) and Erk2^−/−^ (KO) were represented as mean+/− SE under normal diet with tamoxifen injection. (WT, *n* = 9; KO, *n* = 8). (* *p* < 0.05).

WT and Erk2-deficient (Erk2^−/−^, KO) livers under normal chow diet appear similar in terms of liver color, liver sections for hematoxylin and eosin (H&E) staining, Masson’s Trichrome (TRI) staining and the staining of liver fibrosis protein α-SMA and ERK2 (Figure 1a). In H&E and TRI staining, KO livers appear normal as WT livers. The liver weight is similar between WT and Erk2^−/−^ mice. However, the body weight in Erk2^−/−^ mice is slightly lower than WT mice. Therefore, the percentages of liver weight to body weight in Erk2^−/−^ mice were higher than those of WT mice (Figure 1b). The percentages of α-SMA in KO liver sections were similar to that in WT sections. It suggested Erk2^-/-^ livers appear normal as WT liver under the normal chow diet.

### 2.2. Erk2^-/-^ Mice Displayed Less Degree of Liver Fibrosis upon Short-Term Liver Injury

**Figure 2:**
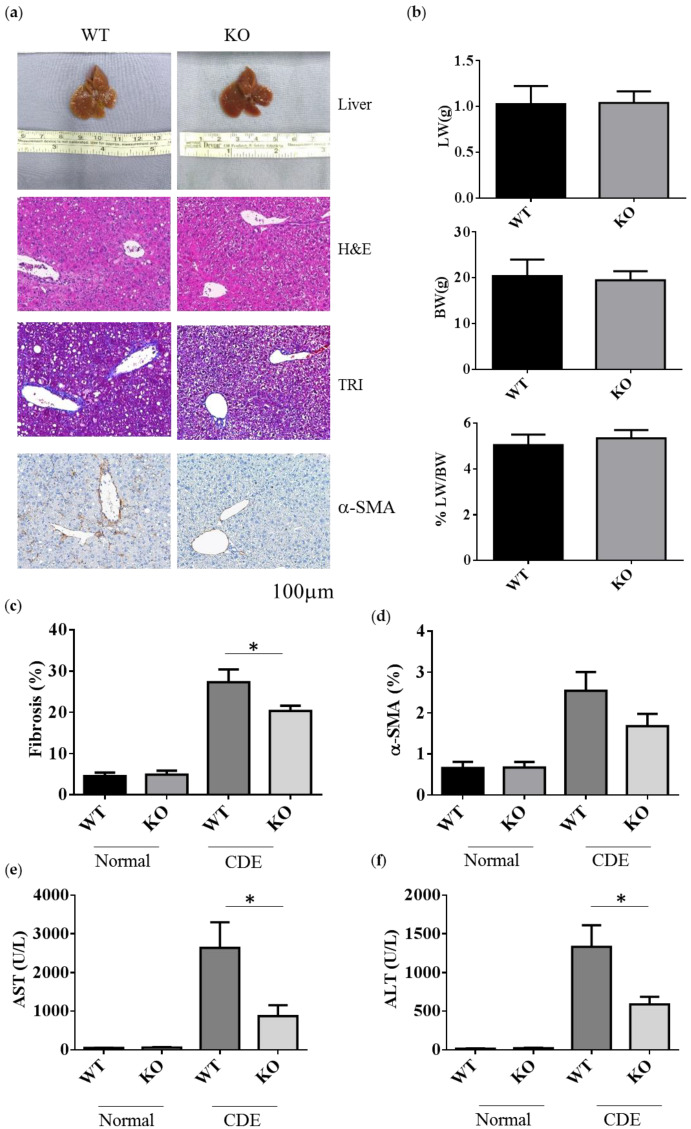
Decreased the degrees of fibrosis in Erk2^−/−^ livers. (**a**) The representative liver histology (H&E staining) or liver fibrosis (TRI staining), alpha-SMA of the WT and Erk2^−/−^ (KO) mice after the CDE treatment; (**b**) The percentages of liver weights to body weights were presented (*n* = 15); (**c**) The percentages of fibrosis from the TRI staining were quantitated by inform software (Normal-WT, Normal-KO *n* = 3, 6 fields each sample, total 18 fields. CDE-WT, CDE-KO *n* = 5, 6 fields each sample, total 30 fields). (*p < 0.05); (**d**) The percentages of α-SMA from the immunohistochemistry staining were quantitated by inform software. (WT, KO *n* = 3, 6 fields each sample, total 18 fields). (**e**,**f**) The serum level of ALT and AST was measured. Normal chew diet: WT (*n* = 12), KO (*n* = 9); CDE diet: WT (*n* = 11), KO (*n* = 11). Data were represented as mean +/− SE and were considered significant if * *p* < 0.05.

To examine the role of Erk2 in liver fibrosis, WT and Erk2^−/−^ (KO) mice were fed with the choline-deficient with supplemented for ethionine (CDE) diet to induce liver fibrosis. WT and Erk2^−/−^ liver were examined for liver weight, body weight and tissues sections for H&E, TRI and α-SMA staining (Figure 2a). After CDE diet treatment, the color of livers turned into brown (Figure 2a first panel). The body weights, liver weights, and the percentages of liver weight to body weight were similar in WT and Erk2^−/−^ mice (Figure 2b). In H&E staining, more infiltrated cells and oval cell hyperplasia were found in WT livers when compared with Erk2^−/−^ livers (Figure 2a second panel). The percentages of fibrosis were calculated by the percentages of collagen area (in blue) to the whole images in the TRI staining. There were no significant differences in the degree of liver fibrosis of WT and Erk2^−/−^ livers under normal chow diet. However, the percentages of liver fibrosis in WT livers was significantly higher than Erk2^−/−^ livers by TRI staining (Figure 2a,c). The percentages of α-SMA in KO liver sections were similar to that in WT sections with the normal chew diet (Figure 2a,d). However, the percentages of α-SMA staining reduced in Erk2^−/−^ livers when compared to WT livers upon the CDE diet (Figure 2a,d).

The degree of liver fibrosis is significantly different in WT and Erk2^−/−^ mice under liver injury by the CDE diet, the levels of aspartate aminotransferase (AST) and alanine aminotransferase (ALT) were examined. Liver cirrhosis could increase the level of AST and ALT. The mouse serum levels of AST and ALT under CDE diet increased 30–50× fold when compared to those under normal chow diet in WT mice (Figure 2e,f). Moreover, Erk2^−/−^ mice displayed significantly lower level of AST and ALT than WT mice under the CDE diet treatment (Figure 2e,f). Taken together, Erk2 plays a role in liver fibrosis upon the short-term liver injury.

### 2.3. Reduction of Gene and Protein Expression Level in Fibrosis Genes alpha-SMA in Erk2^−/−^ Livers

**Figure 3:**
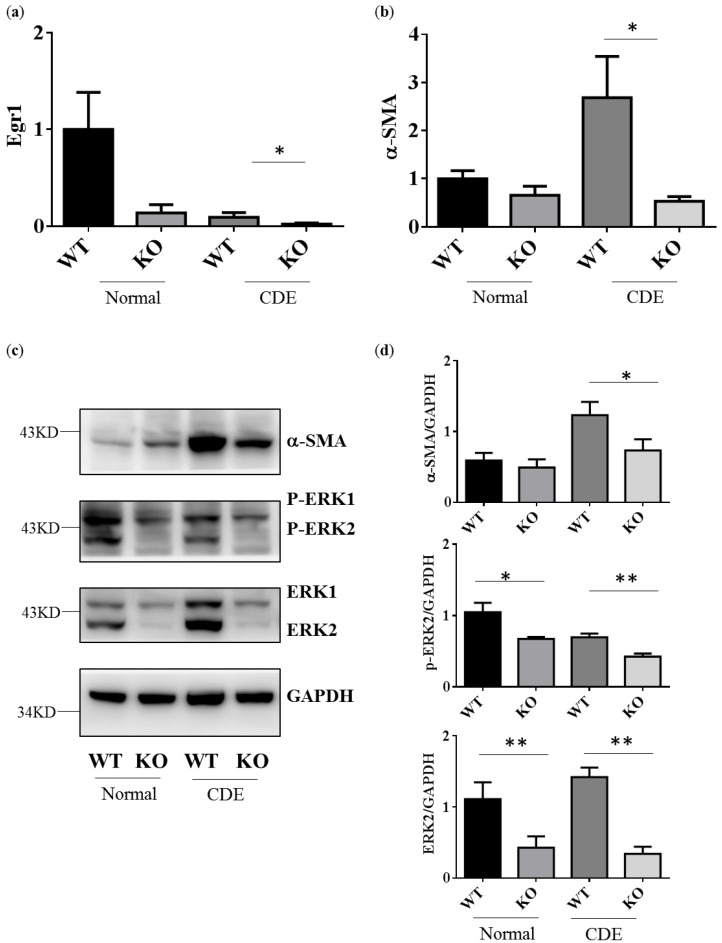
Erk2^−/−^ (KO) livers express less cirrhosis gene α-SMA than WT livers. Gene expression of Egr1 (**a**), alpha-SMA (**b**) were determined by QPCR. Data were normalized by the average of expression level in WT under the normal chew diet (WT, normal) as 1. Data were represented as mean +/− SE and were considered significant if * *p* < 0.05; ** *p* < 0.01. WT (*n* = 4), KO (*n* = 4); (**c**) WT and Erk2^−/−^ livers were subjected to Western Blotting and analyzed for protein expression in alpha-SMA, phospho-ERK (P-ERK1 and P-ERK2), ERK (ERK1 and ERK2) and loading control glyceraldehyde-3-phosphate dehydrogenase (GAPDH); (**d**) Protein expression were quantized by Image J and normalized by the expression of GAPDH. The relative protein expression level was shown. (WT *n* = 4; KO *n* = 4; * *p* < 0.05; ** *p* < 0.01).

Egr1 is one of downstream genes of ERK signaling. As expected, Egr1 gene expression would be down-regulated in Erk2^−/−^ (KO) livers when compared to WT livers (Figure 3a). CDE diet could induce oval cells proliferation and liver cirrhosis-related gene expression. Alpha-SMA (Asma) expression is positively correlated with the degree of fibrosis. It is one of specific markers for liver fibrosis and hepatocyte stellate cells (HSCs) activation. The level of alpha-SMA is higher in WT livers under the CDE diet when compared to WT under the normal diet (Figure 3b). Erk2^−/−^ (KO) mice express less level of liver cirrhosis genes than WT (Figure 3b). In addition, there is a significant reduction in the protein level of alpha-SMA in Erk2^−/−^ livers when compared to WT livers under the CDE diet treatment (Figure 3c,d). As expected, the protein level of ERK2 and phos-ERK2 was significantly lower in Erk2^−/−^ livers in the comparison with WT livers (Figure 3c,d).

### 2.4. Reduction of Cell Proliferation in Erk2^−/−^ Livers upon Liver Fibrosis

**Figure 4:**
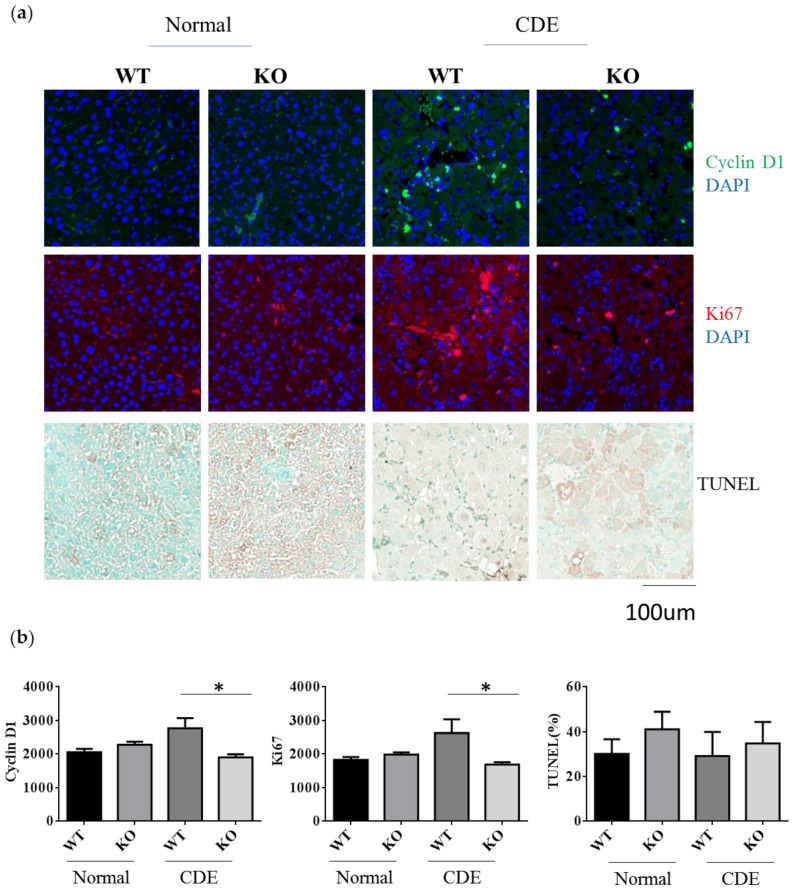
Reduction of cell proliferation in Erk2^−/−^ (KO) mice under the CDE diet. (**a**) WT and Erk2^−/−^ liver sections were stained with cyclin D1 and ki-67 with DAPI staining for nucleus. Liver sections were stained with TUNEL assay kit HRP-DAB; (**b**) The average intensities of cyclin D1 and ki-67were measured by MetaXpress analysis software. (WT, KO *n* = 3, 6 fields each sample, total 18 fields). The percentages of TUNEL positive area (brown) were quantitated by inform software (WT, KO *n* = 3, 6 fields each sample, total 18 fields; * *p* < 0.05).

Since there was a significant difference in the degree of fibrosis in WT and Erk2^−/−^ (KO) livers, it would be interesting to investigate whether the differences of cell proliferation or apoptosis are significant between two groups. Cyclin D1 plays an important role in cell cycle and ki-67 labels proliferative response. The average intensities of Cyclin D1 and Ki-67 were similar in WT and KO under the normal diet (Figure 4a,b). There was a significant reduction of Cyclin D1 and Ki-67 in KO livers when compared to WT livers under the CDE diet (Figure 4a,b). The terminal deoxynucleotidyl transferase dUTP nick end labeling (TUNEL) stains for the apoptotic cells. The percentages of TUNEL positive area were similar in WT and Erk2^−/−^ (KO) livers (Figure 4a,b). Although the percentages of TUNEL positive area are higher in KO livers, it is not significant. Taken together, Erk2 deficiency could lead to significant reduction in liver cell proliferation.

### 2.5. Reduced Activated T Cells in Erk2^−/−^ (KO) upon the CDE Diet Treatment

**Figure 5:**
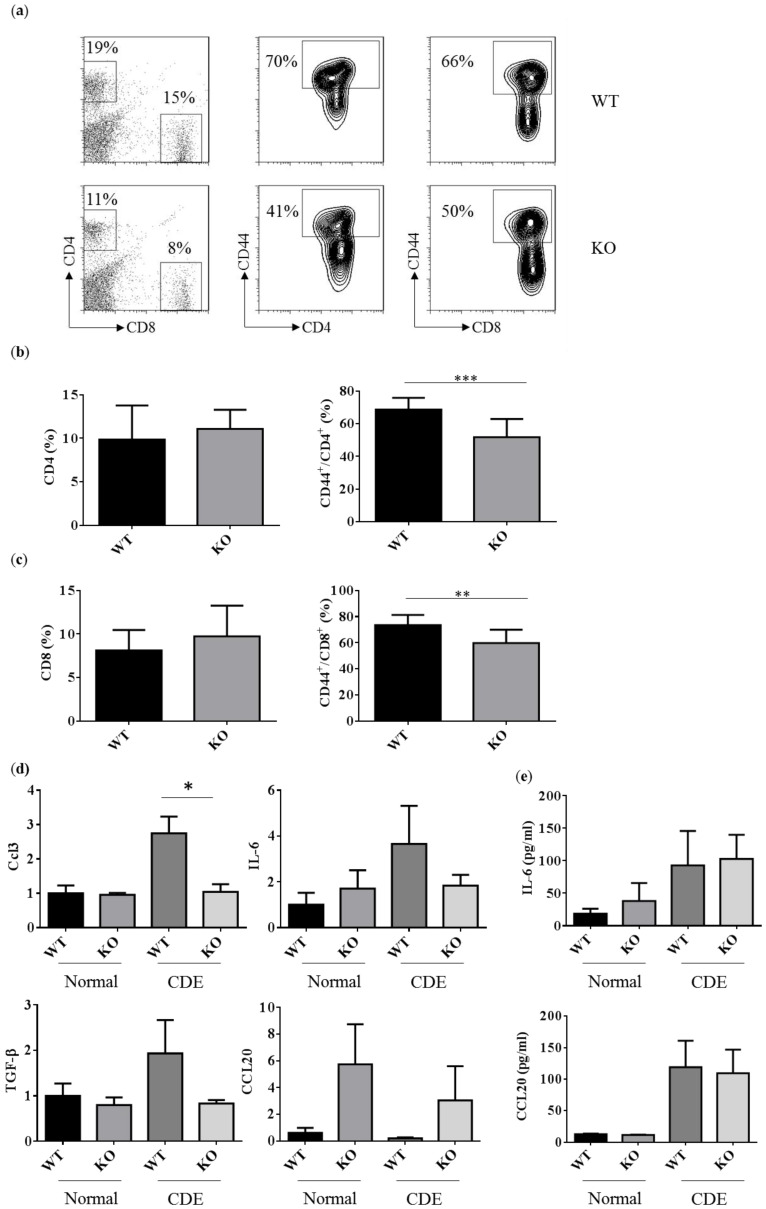
Reduced activated T cells in Erk2^−/−^ (KO) upon the CDE diet treatment. (**a**) hepatic lymphocytes from WT and Erk2^−/−^ (KO) livers were stained for T cells (CD4 or CD8) and activated markers (CD44). The percentages of CD4, CD8, CD44^+^/CD4^+^ and CD44^+^/CD8^+^ T cells were shown in representative WT and Erk2^−/−^ (KO) liver; (**b**) The percentages of CD4 T cells and CD44^+^/CD4^+^ T cells were represented as mean +/− SE (WT *n* = 12; KO *n* = 12); (**c**) The percentages of CD8 T cells and CD44^+^/CD8^+^ T cells were represented as mean +/− SE (WT *n* = 12; KO *n* = 12). The differences between WT and KO were considered significant if *p* < 0.05. (** *p* < 0.01; *** *p* < 0.001); (**d**) Gene expression of Ccl3, IL-6, TGF-β and Ccl20 were determined by QPCR. Data were normalized by the average of expression level in WT under the normal chew diet (WT, normal) as 1. Data were represented as mean +/− SE and were considered significant if * *p* < 0.05; ** *p* < 0.01. WT (*n* = 4), KO (*n* = 4); (**e**) the serum level of IL-6 and CCL20 was measured. Normal chew diet: WT (*n* = 6), KO (*n* = 6); CDE diet: WT (*n* = 7, KO (*n* = 7). Data were represented as mean +/− SE and were considered significant if * *p* < 0.05.

The T cell responses were examined to address the degree of liver inflammation upon the CDE diet treatment. The percentages of CD4 and CD8 T cells were about 11–19% and 8–15% in WT and Erk2^−/−^ livers (Figure 5a left panel). There were no significant differences in the percentages of CD4 and CD8 T cells in WT and Erk2^−/−^ livers (Figure 5b,c left panel). The percentages of CD44+ in CD4 T cells were about 70% in WT livers and 41% in Erk2^−/−^ (KO) livers (Figure 5a middle panel). There is a significant reduction in the percentages of activated CD4 T cells (CD4^+^CD44^+^) in KO livers (Figure 5b). In addition, the percentages of activated CD8 T cells (CD8^+^CD44^+^) reduced significantly in KO livers when compared to WT livers (Figure 5a right panel, c). It suggested that less activation of T cell responses in Erk2^−/−^ (KO) livers upon liver fibrosis.

In addition, the immune responses in liver fibrosis and the degree of liver fibrosis could be regulated by the cytokines or chemokines. CCL3 could increase proliferation and migration of hepatic stellate cells (HSCs). There was a significant reduction in CCL3 expression in KO livers compared to WT liver upon the CDE diet (Figure 5c upper left panel). However, the level of CCL3 is too low to be detected in mouse serum. Besides, IL-6 induces hepatic inflammation and collagen synthesis. The level of IL-6 increased in the CDE diet group when compared to the normal chow diet group (Figure 5c upper right panel, 5d). However, there were no significant differences in WT and KO livers or serum. HSCs and Kupffer cells (KCs) can express TGFβ to inhibit T cell activation and proliferation. There were no significant differences in gene expression level of TGFβ in WT and KO livers upon the CDE diet. Moreover, CCL20 mediates the process for liver fibrosis. The gene expression level of CCL20 is lower than other cytokines such as CCL3, IL-6, TGFβ. Although the relative CCL20 gene expression level is higher in KO livers, there were no significant differences between WT and KO livers (Figure 5c bottom right panel). Although the serum levels of CCL20 were higher in the CDE diet group than normal chow diet group, there were no differences between WT and KO serum (Figure 5d).

### 2.6. Differentially Expression Genes (DEGs) of WT and Erk^−/−^ Livers upon Liver Injury

**Figure 6:**
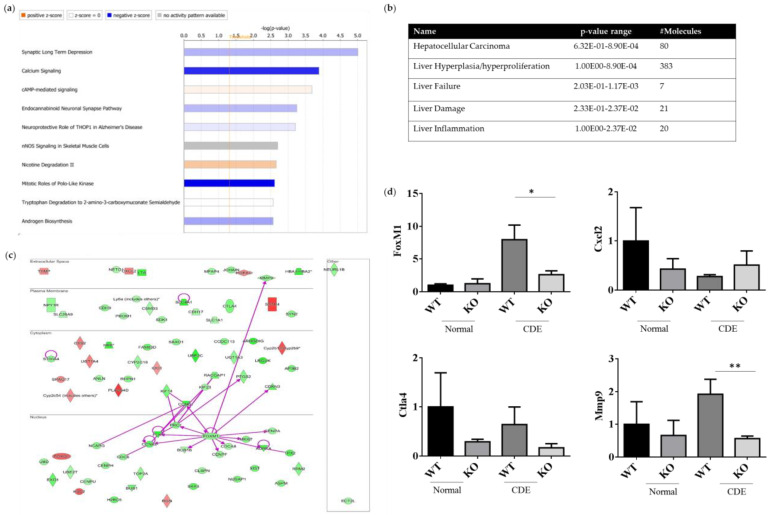
ERK2-regulated differential expression genes (DEGs) in canonical signaling pathways. DEGs from WT and Erk2^−/−^ livers under the CDE diet treatment (each group, *n* = 5) were subjected to the analysis of canonical signaling pathways. (**a**) Top-10 enriched canonical signaling pathways identified by Ingenuity Pathway Analysis. Orange, activated; blue, inhibited; gray, no activity pattern available. (Positive z-score, activation; negative z-score, inhibition). (**b**) Erk2-dependent DEGs in hepatotoxicity. (**c**) 80 DEGs in HCC were represented in red (up-regulation) and green (down-regulation). The direct interactions were shown in purple solid arrows. Molecules are shown in their locations such as extracellular space, plasma membrane, cytoplasm, nucleus and other. (**d**) Gene expression of FoxM1, Cxcl2, Ctla4, and Mmp9 were determined by QPCR. Data were normalized by the average of gene expression level in WT under the normal chew diet (WT, normal) as 1. Data were represented as mean +/− SE and were considered significant if *p < 0.05; ** *p* < 0.01. WT (*n* = 4), KO (*n* = 4).

Both the gene expression profiles WT and Erk2^−/−^ (KO) livers under the CDE diet treatment were subjected to next-generation sequencing analysis for the whole gene expression profiles. The top 10 canonical signaling pathways were identified from differential expression genes (DEGs) (Figure 6a). MAPK1 (ERK2) is involved in the top 2 to 4 canonical signaling pathways, including calcium signaling, cAMP-mediated signaling and endocannabinoid Neuronal Synapse Pathway (Table S1). ERK2 deficiency could lead to reduction in the canonical pathway mitotic roles of Polo-like kinase for cell cycle and cell division. In addition, DEGs of WT and Erk2^−/−^ livers under the CDE diet were further classified in hepatotoxicity. There were 80 molecules in hepatocellular carcinoma with a *p*-value form 6.32E-01 to 8.90E-04 (Figure 6b and Table S2). Moreover, 383 DEGs are found in liver hyperplasia/hyperproliferation and 7–21 DEGs were identified in liver failure, liver damage and liver inflammation (Figure 6b and Table S2).

The direct interactions of DEGs for HCC were represented (Figure 6c). 66 genes were down-regulated in green whereas 14 genes were up-regulated in red, when comparing the expression profiles of Erk2^−/−^ and WT livers under liver fibrosis. Our data suggested FoxM1 is down-regulated in liver injury in the absence of ERK2. Previous studies demonstrated ERK signaling could induce Forkhead Box M1 (FoxM1) to promote trophoblast cell invasion and mediate TGF-β-induced EMT in non-small cell lung cancer [29,30]. FoxM1 directly interacts with many HCC-related molecules such as cyclin B1(CNNB1), protein regulator of cytokinesis (PRC1), polo-like kinase 1 (PLK1), cyclin A2 (CCNA2), BUB mitotic checkpoint serine/threonine kinase B (BUB1B), centromere protein F (CENPF), cell division cycle associated protein 8 (CDCA8), aurora kinase A (AURKA), marker of proliferation Ki-67 (MKI67), centromere protein A (CENPA) and MMP9 (Figure 6c). EMT marker MMP 9 is down-regulated in Erk2^−/−^ livers. Moreover, the stem cell marker CD133 (PROM1) is also down-regulated in Erk2^−/−^ livers. On the other sides, up-regulated genes include somatostatin receptor 4 (SSTR4), phospholipase A2 group IVD (PLA2G4D), cytochrome P450 family 2, subfamily b, polypeptide 9 (Cyp2b9) etc. The gene expression of FoxM1, Cxcl2, Ctla4 and Mmp9 were confirmed by the quantitative polymerase chain reaction (qPCR). In addition, the gene expression levels of Cxcl2, Ctla4 and Mmp9 by Q-PCR is to confirm the next generation sequencing (NGS) data. It has similar results that Erk2^−/−^ (KO) livers reduced the expression of FoxM1, Ctla4, Mmp9 and increased the expression of Cxcl2 when compared to WT liver upon the CDE diet (Figure 6d).

## 3. Discussion

Liver fibrosis/cirrhosis is associated with aberrant apoptosis of hepatocytes, collagen formation and hepatic lymphocyte inflammation [31,32]. ERK signaling pathway modulates different cellular responses in liver fibrogenesis of hepatic myofibroblasts [33]. ERK2 plays a more important role than ERK1 because ERK2 expression in livers display normal function with ERK1 deficiency [13,14]. ERK1 deficiency mice display normal proliferation with the normal protein level of the cyclin D1, Cdk1 and BrDU [13]. It demonstrated ERK1 is dispensable for hepatocyte replication. It is correlated with the study that ERK1^−/−^ mice are viable and have normal sizes [34]. However, the inhibition of ERK2 expression abolished the DNA synthesis. ERK2 could have a positive role in controlling cell proliferation. Therefore, this study focused on the role of ERK2 in liver fibrosis.

Erk2^−/−^ mice display less degree of liver fibrosis in the comparison to WT mice in terms of the expression of fibrosis markers alpha-SMA, collagen staining by TRI and liver enzymes (AST, ALT). Alpha-SMA is a detective marker for the early stage fibrosis [35]. The expression of phos-ERK is positively correlated with the expression of alpha-SMA in HCC patients [36]. In hepatic cell line, apelin could activate ERK signaling to increase alpha-SMA, collagen I and cyclin D1 [37]. ERK signaling could activate type I collagen in fibroblasts [38]. It is agreed with our study that ERK2 plays an important role in regulating the liver fibrosis. In liver fibrosis model (Methionine-choline deficient, MCD diet), the levels of ALT AST of mice were about 500 and 900 U/L [39]. In this study, the levels of ALT were over 1000 U/L in WT mice under the CDE diet treatment. It could depend on the mice variance. Although the levels of AST and ALT in our study were higher, the differences between WT and KO were still significant. Liver fibrosis is involved in the imbalance of cell proliferation and apoptosis. ERK2 signaling is required for cell proliferation and survival [40]. In this study, ERK2 deficient livers express less cyclin D1 and Ki-67. It suggested less proliferation occurs in ERK2 deficient liver upon the process of fibrosis. It could be a reason that ERK2 deficient mice display less degree of liver fibrosis.

In addition, hepatic inflammatory cells include activated T lymphocytes such as CD4+CD44+ and CD8^+^CD44^+^ cells [41]. ERK2^−/−^ hepatic lymphocytes display less the percentages of CD44 expression in the comparison to WT upon liver fibrosis. CD44 is a key marker for the hepatic inflammation and strongly up-regulated in non-alcoholic steatohepatitis (NASH) patients [42]. Moreover, CD44 is a cancer stem cell marker and mediates signaling pathways for tumor differentiation, invasion, and metastasis [43]. The CD44 and CD133 expression increased with the grades of inflammation and stages with fibrosis upon virus infection [44]. The expression of CD133 was down-regulated in Erk2^−/−^ livers when compared to WT livers in this study.

In addition, the cytokines and chemokine regulates liver fibrosis and immune responses. CCL3 is a mediator of experimental liver fibrosis [45]. CCL3^-/-^ mice less stellate cell activity and express less α-SMA. There was a significant reduction in Ccl-3 gene expression in KO livers. It is similar ERK2^−/−^ and CCL3^−/−^ mice had less degree of liver fibrosis. However, the level of CCL3 could not be detected in WT and KO mice serum. IL-6 induces hepatic inflammation and collagen synthesis [46,47]. HSCs and KCs can express TGFβ to inhibit T cell activation and proliferation. In addition, CCL20 is up-regulated in liver fibrosis diseases [48]. However, there were no significant differences in IL-6, TGFβ, CCL20 in WT and Erk2^−/−^ livers.

Several DEGs of HCC markers were down-regulated in Erk2^−/−^ mice under the liver fibrosis. HCC displays the heterogeneity of histopathological characteristics [49]. Therefore, it is necessary to identify the molecular mechanisms of ERK2-dependent biomarkers in the process of liver cirrhosis, even to HCC. In particular, the expression of FoxM1 was reduced in Erk2^−/−^ livers in the comparison with WT livers. FoxM1 could promote cell proliferation, EMT and metastasis in human HCC [50,51,52]. In mouse model, FoxM1 drives inflammation responses for liver fibrosis and hepatocarcinogenesis [53]. It is possible that FoxM1 is down-regulated to reduce inflammation responses in absence of ERK2. Therefore, Erk2^−/−^ livers display less degree of liver fibrosis than WT livers.

MMP9 belongs to matrix metalloproteinase family involved in liver injury, repair, and fibrosis [54]. The balance between MMP and extracellular matrix (ECM) protein is important for liver homeostasis. MMP9 expression is detected in the early stages of hepatic fibrogenesis. The X protein of HBV could induce MMP9 expression through ERK and PI3K pathway in HCC [55]. Our study demonstrated ERK2 signaling could regulate MMP9 expression in liver fibrosis mouse model. Moreover, CD133 (PROM1) is a progenitor cell marker in liver and cancer stem cell marker in HCC [56,57]. CD133 positive HCC cell clone have constitutively express the phosphorylation of ERK1/2 [58]. It suggested less CD133 expression were found in liver fibrosis in absence of ERK2 signaling. If altering ERK signaling could reduce the degree of liver fibrosis, it would slow down the process from liver fibrosis to HCC. Therefore, our study provides the new insight that altering ERK signaling pathway by ERK2 deficiency alone could reduce liver fibrosis and inflammatory responses.

## 4. Materials and Methods

### 4.1. Mice and CDE Diet Treatment

The Erk2f/f is generated by Dr. April Fischer and Dr. Stephen M. Hedrick [59,60]. The ERCre mice is kindly provided by Dr. Luwig [61]. The Erk2f/f. ERCre were generated by the cross of Erk2f/f mice and ERCre mice. All animal experiments were approved IACUC of Far Eastern Memorial Hospital (No. 105-01-37-C2). 7–8 weeks old wild-type and Erk2f/f. ERCre mice were injected i.p. with 2mg tamoxifen (Sigma, St. Louis, MO, USA) for five consecutive days to induce Erk2 deletion in Erk2f/f. ERCre mice as Erk2^−/−^ mice. Livers were harvested 6 weeks after normal chow diet or choline-deficient (#518753, DYETS Inc., Bethlehem, PA, USA) ethionine (Sigma)-supplemented (CDE) diet.

### 4.2. Histology

Liver tissue were fixed in formalin and embedded in parafilm wax. Liver sections were stained with hematoxylin and eosin (H&E) according to the standard protocol. Masson’s Trichrome (TRI) Stain was used to examine the degree of liver fibrosis due to increasement of collagen. TRI stain represents three acidic dyes: aniline blue to stain collagen, acid fuchsin for cytoplasm or muscle (bright-red) and orange G for red blood cells. For immunohistochemistry staining, liver tissue sections were stained with primary antibody against alpha-SMA (Abcam #124964, Cambridge, UK). The secondary antibody conjugated with HRP (Abcam #406401) and DAB substrate kit (Abcam #ab64238) were used to visualize the signal. To measure hepatocyte apoptosis, the TUNEL assay was performed on liver paraffin section label the 3-end of fragmented DNA by an apoptosis detection kit according to the manufacturer’s instructions (Abcam #206386). All images were acquired on a LUMAR12 inverted microscope (Olympus Corp., Tokyo, Japan) with Nuance Multispectral image acquisition software system (PerkinElmer, Waltham, MA, USA). The images were quantified by inform software (PerkinElmer). Foe immunofluorescence staining, liver tissues were stained for cyclin D1 (Millipore Corp., Burlington, MA, USA) followed by the secondary antibody conjugated with fluorescein isothiocyanate (FITC) and Ki-67-e615 (ebioscience Inc., San Diego, CA, USA). All images were acquired on the ImageXpress Micro 4 system and analyzed MetaXpress High-Content Image Acquisition and Analysis Software (Molecular Devices LLC., San Jose, CA, USA).

### 4.3. Serum Analysis of ALT and AST

The serum levels of ALT and AST were measured by the Hitachi 7080 autobiochemical analyzer (Hitachi Ltd., Tokyo, Japan) in the National Laboratory Animal Center, Taiwan. The mouse serum levels of IL-6 and CCL20 were measured by the ELISA kit (Biolegend Inc., San Diego, CA, USA)

### 4.4. RNA Isolation and Quantitative Gene Expression

Total RNA was isolated from liver cells using RNeasy plus mini kit (Qiagen Inc., Redwood city, CA, USA) according to the manufacture’s protocol. Total RNA was reversely transcripted to cDNA by high capacity cDNA RT kit (Applied Biosystems Inc., Foster, CA, USA). The mRNA expression was under analysis using real-time PCR machine Roche LightCycler480 (Roche Applied Science, Mannheim, Germany). The level of target gene expression was obtained by the normalization to the endogenous gene (GAPDH).

### 4.5. Western Blotting

Cell lysates were collected by lysis buffer on ice and the protein concentrations was determined with the BCA assay (PierceTM #23227, ThermoFisher Scientific, Rockford, IL, USA). Protein samples were loaded into gels and electrophoresed on 10% gels depends on the molecular weight of protein. Separated proteins gels were transferred to PVDF membranes and then blocked with 5% BSA blocking buffer for 30 min prior to primary antibody incubation. The primary antibody was used at room temperature for 2 h including a rabbit polyclonal antibody against mouse ERK1/2 (Cell signaling #9102, Danvers, MA, USA), Phos-ERK (Cell signaling #9101), alpha-SMA (Abcam#124964) and GAPDH (Abcam #181602) as an internal control. The secondary antibodies with HRP conjugated were used and detected by using enhanced chemiluminescence (ECL). Images were captured and analyzed densitometrically by LAS 3000 imaging system (Fujifilm Corp., Tokyo, Japan). Software Image J was used to quantify the protein expression of alpha-SMA and ERK2 normalized by GAPDH.

### 4.6. Flow Cytometry

Hepatic lymphocytes were subjected to flow cytometry analysis. Cells were stained for Fluorescent-labeled antibodies against CD4, CD8 and CD44 (Biolegend Inc., San Diego, CA, USA). Samples were subjected and collected from the flow cytometer FACS Calibur (BD Sciences, San Jose, CA, USA). Data were analyzed by the Flowjo software (Tree Star Inc., Ashland, OR, USA).

### 4.7. RNA Sequencing

Total RNA was isolated from liver cells using the RNeasy plus mini kit (Qiagen, Redwood city, CA, USA) according to the manufacture’s protocol. RNA was fragmented for RNA libraries by using 200–500 ng of total RNA according to TrueSeq Stranded mRNA kit (Illumina, San Diego, CA, USA). For sequencing, mRNA library was subjected to HiSeq 3000/4000 PE Cluster kit and analyzed by HiSeq4000 (Illumina). Post sequencing base calling and adaptor trimming were used by the Trimmomatic. Data mapping were analyzed by the Bowtie 2 software (Johns Hopkins University, Baltimore, MD, USA) and gene expression were analyzed by RSEM software. Gene reads were normalized by fragments mapped per kilo base length of the transcript per million reads (FPKM) method. EBseq software (Bioconductor, R package) and Ingenuity Pathway Analysis (IPA, Qiagen) software was used to analyze the differential gene expression (DGE) from WT and Erk2^−/−^ livers. DGEs were selected from the gene expression of fold change (KO/WT) is less 0.5-fold and over 2-fold.

### 4.8. Statistical Analysis

Statistical analysis and graphs were created by GraphPad Prism software (GraphPad software, San Diego, CA, USA). All the data was reported as mean ± standard error. Comparisons between different groups were performed using the *t*-test. *p* < 0.05 was considered statistically significant.

## 5. Conclusions

ERK2^−/−^ mice display the reduction in the degree of liver fibrosis and less inflammation under liver fibrosis mouse model. ERK2 signaling pathway regulates the progression liver fibrosis through multiple HCC-related biomarkers such as FoxM1, MMP9, CD133 etc. ERK2 deficiency could lead to reduction in cell proliferation in liver fibrosis. In conclusion, altering ERK signaling pathway by ERK2 deficiency could reduce liver fibrosis and inflammatory responses.

## Supplementary Materials

Supplementary materials can be found at https://www.mdpi.com/1422-0067/21/11/3796/s1. Table S1. Gene list of Erk2 regulated DEGs in the top 10 canonical signaling pathways; Table S2. Gene list of Erk2 regulated DEG in hepatotoxicity.

## Abbreviations

HCC	Hepatocellular carcinoma
ERK	Extracellular signal-regulated kinase 2
CDE	Choline-deficient, ethionine-supplemented
HSC	Hepatocyte stellate cell
alpha-SMA	Alpha smooth muscle
MAPK	Mitogen-activated protein kinase
RKIP	RAF kinase inhibitory protein
HBV	Hepatitis B virus
EMT	Epithelial-mesenchymal transition
H&E	Hematoxylin and eosin
TRI	Masson’s Trichrome
LW	Liver weight
BW	Body weight
CDE	Choline-deficient with supplemented for ethionine
AST	Aspartate aminotransferase
ALT	Alanine aminotransferase
GAPDH	Glyceraldehyde-3-phosphate dehydrogenase
TUNEL	Terminal deoxynucleotidyl transferase dUTP nick end labeling
KCs	Kupffer cells
DEGs	Differential expression genes
FoxM1	Forkhead Box M1
CNNB1	Cyclin B1
PRC1	Protein regulator of cytokinesis
PLK1	Polo-like kinase 1
CCNA2	Cyclin A2
BUB1B	BUB mitotic checkpoint serine/threonine kinase B
CENPF	Centromere protein F
CDCA8	Cell division cycle associated protein 8
AURKA	Aurora kinase A
MKI67	Marker of proliferation Ki-67
CENPA	Centromere protein A
SSTR4	Somatostatin receptor 4
PLA2G4D	Phospholipase A2 group IVD
Cyp2b9	Cytochrome P450 family 2, subfamily b, polypeptide 9
qPCR	quantitative polymerase chain reaction
NGS	Next generation sequencing
MCD	Methionine-choline deficient
NASH	Non-alcoholic steatohepatitis
ECM	Extracellular matrix
FITC	Fluorescein isothiocyanate
