# Supplementary material for: Liver Fibrosis and Inflammation under the Control of ERK2
Source: Int J Mol Sci. 2020 May 27;21(11):3796. doi: 10.3390/ijms21113796 (PMC7312875; doi:10.3390/ijms21113796)
Supplement: Supplementary file 1 [file ijms-21-03796-s001.pdf]

Table S1. Gene list of Erk2-regulated DEGs in the top-10 canonical signaling pathways

Canonical Pathways	z-score	Molecules
Synaptic Long Term Depression	-0.853	CACNA1B,CACNA1E,CACNA2D4,CACNB2,CACNG8,CRHR1,GAD1,GNAZ,GRIA4,GRM1,GRM8,Gucy1b2,GUCY2D,MAPK1,NOTUM,PLA2G2D,PLA2G2E,PLA2G4D,PLA2G5,PLCH2,PRKD1,PRKG2,RYR1
Calcium Signaling	-2.357	AKAP5,ATP2A1,ATP2B3,CACNA1B,CACNA1E,CACNA2D4,CACNB2,CACNG8,CALML5,CASQ1,CHRNA10,CHRNA4,CHRNB4,CHRNE,GRIA4,GRIN2C,MAPK1,MYL1,RYR1,TNNI2,TNNI3,TRPM8
cAMP-mediated signaling	0.218	ADORA3,ADRA2C,ADRB3,AKAP4,AKAP5,APLNR,CALML5,CCR4,CHRM1,CHRM2,CHRM4,CNGA2,CNGB3,CRHR1,GPR17,GRM8,HTR7,MAPK1,NPY1R,OPRL1,PDE6G,PDE6H,TULP2
Endocannabinoid Neuronal Synapse Pathway	-0.775	CACNA1B,CACNA1E,CACNA2D4,CACNB2,CACNG8,GRIA4,GRIN2C,GRM1,KCNJ3,KCNJ9,MAPK1,NOTUM,PLCH2,PTGS2,RIMS1
Neuroprotective Role of THOP1 in Alzheimer's Disease	-0.277	CMA1,CTSG,GNRH1,HGFAC,HLA-A,KLK3,MMP9,PRSS12,Prss30,Prss34,PRSS42P,PRSS53,PRTN3,TPSG1
nNOS Signaling in Skeletal Muscle Cells	NaN	CACNA1B,CACNA1E,CACNA2D4,CACNB2,CACNG8,CALML5,RYR1
Nicotine Degradation II	1	Aox4,CYP1A1,CYP2C18,CYP3A5,Fmo6,UGT1A3,UGT1A4,UGT2A3,UGT3A1
Mitotic Roles of Polo-Like Kinase	-2.828	CCNB1,CCNB2,CDC20,CDC25C,CDK1,KIF11,KIF23,PLK1,PRC1
Tryptophan Degradation to 2-amino-3-carboxymuconate Semialdehyde	NaN	AFMID,HAAO,IDO1
Androgen Biosynthesis	-1	HSD17B14,HSD17B3,HSD3B2,Hsd3b4 (includes others)

* A positive z-score indicates a predicted activation, a negative z-score indicates a predicted inactivation of the enriched pathway. NaN: a z-score cannot be calculated for all ingenuity canonical pathway.

Table S2. Gene list of Erk2-regulated DEG in hepatotoxicity

Name	Molecules
Hepatocellular carcinoma	ANLN,AP3B2,ARHGDIG,ASPM,AURKA,BUB1,BUB1B,CCDC113,CCNA2,CCNB1,CDC6,CDCA8,CDH17,CDH9,CDKN3,CENPA,CENPF,CENPH,CENPU,CES2,CLSPN,CSMD3,CTLA4,CXCL2,Cyp2b13/Cyp2b9,CYP2C18,Cyp2c54,ECT2L,EXO1,FAM83D,FOXM1,FOXQ1,H2BC5,H3C2,HBA1/HBA2,HBB,HGFAC,IDO1,JCHAIN,KIF14,KIF23,LRGUK,LTA,Ly6a,MFAP4,MKI67,MMP9,NCAPG,NETO1,NEURL1B,NPY1R,NUSAP1,PLA2G4D,PLK1,PRC1,PROM1,PTGS2,RACGAP1,RGN,RHPN1,RRM2,S100A4,SAXO1,SDK1,SKA3,SLC1A1,SLC26A9,SLC4A1,SPAG17,SSTR4,SYN2,TOP2A,TPX2,TYMP,UBD,UBE2C,UBE2T,UGT1A3,UGT1A4,XIST
Liver Hyperplasia/ Hyperproliferation	ABCC8,ABCG4,ACSL6,ACTG2,ADAD2,ADAM2,ADRA2C,AMN,AMPD1,ANK1,ANKMY1,ANKS1B,ANKUB1,ANLN,ANO7,AP3B2,ARHGDIG,ARNT2,ASB11,ASB15,ASPM,ASTN1,ATP1A3,ATP4A,AURKA,AURKB,BIRC5,BRINP2,BUB1,BUB1B,C17orf97,C21orf62,CA12,CA4,CACNA1B,CACNA1E,CACNA2D4,CACNB2,CAMP,CCDC113,CCDC13,CCDC38,CCDC65,CCL2,CCNA2,CCNB1,CCNB2,CCR6,CCR9,CCT6B,CD5,CDC20,CDC6,CDCA2,CDCA3,CDCA8,CDH17,CDH9,CDHR1,CDKN3,CENPA,CENPE,CENPF,CENPH,CENPU,CES2,CHRM1,CHRM2,CHRM4,CHRNA10,CHRNA4,CHTF18,CISH,CKAP2L,CLCA4,CLSPN,CLVS2,CMA1,CNGA2,CNGB3,CRP,CRTAM,CSMD1,CSMD3,CSPG5,CSRP3,CTLA4,CTNNA3,CUX2,CXCL2,Cyp2b13/Cyp2b9,CYP2C18,Cyp2c54,CYP3A5,DCX,DDN,DDX4,DDX43,DEPDC1,DGKK,DIAPH3,DLEC1,DLGAP1,DMBT1,DMKN,DNAH2,DNAH6,DNASE1,DPEP3,DSC3,DUSP27,ECT2L,EEF1A2,EFCAB5,ENOX1,EPHA6,EPPIN,EPX,EXO1,EYA1,FAM131B,FAM166A,FAM205A,FAM83D,FANCI,FAT3,FBP2,FBXO47,FCER1A,FDPS,FOXM1,FOXQ1,FRMPD1,GABRB2,GAMT,GBP3,GDAP1L1,GDF11,GJB5,GLIS1,GLT6D1,GPR156,GPR17,GPR25,GPR75,GRHL3,GRIA4,GRIN2C,GRM8,GSTA5,GUCY2D,H2AC20,H2BC3,H2BC5,H2BU1,H3C2,H3C8,HBA1/HBA2,HBB,HCRTR2,HEMGN,HFM1,HGFAC,HMMR,HOXB7,HSD11B2,HSD3B2,IDO1,IL1R2,IL23R,INSIG1,INSM1,IQGAP3,JCHAIN,KBTBD12,KCNA7,KCNIP2,KCNK12,KCNK3,KCNT1,KIF14,KIF18B,KIF23,KIFC1,KNTC1,LIN28A,LINGO4,LRGUK,LRIT1,LRP8,LRRC55,LRRC73,LRRTM3,LRTM2,LTA,Ly6a,MALRD1,MARCHF11,MATN4,MEGF11,MFAP4,MKI67,MMP16,MMP9,MORC1,MORN5,MOXD1,MYBPC3,MYLPF,MYO16,MYO18B,MYO3B,MYOC,MYOCD,MYOM2,NAALADL1,NANOG,NCAPG,NEK2,NETO1,NEURL1B,NKAIN3,NLRP5,NOTUM,NOVA1,NPY1R,NRG2,NUSAP1,OLFML2A,OPRL1,OR11A1,OR1E2,OR5M11,OR5V1,PADI3,PALM3,PBLD,PCDH11X,PCDH9,PCDHA5,PCDHAC2,PEG3,PGR,PI15,PI16,PIF1,PKHD1L1,PKP1,PLA2G4D,PLK1,POU4F1,PPM1E,PPP1R42,PRAMEF12,PRC1,PRG2,PRKD1,PRKG2,PRND,PRODH2,PROM1,PRR22,PRR29,PRRT4,PTGS2,PTPRN,PTPRR,QRICH2,RACGAP1,RBM44,RDH16,RGN,RHO,RHPN1,RIMS1,RPL32,RPL3L,RPTN,RRM2,RTKN2,RYR1,S100A4,S100A6,SAMD12,SAP25,SAXO1,SCN8A,SDK1,SEMA4F,SERPINB8,SERTM1,SEZ6L,SFMBT2,SGCA,SHC3,SHISA6,SKA3,SLC10A4,SLC1A1,SLC1A7,SLC22A14,SLC22A25,SLC26A7,SLC26A9,SLC27A5,SLC4A1,SLC5A2,SLC6A17,SLC6A18,SLC6A20,SLC7A3,Slco1a1,SMPX,SNTG2,SNX31,SORCS3,SOX11,SOX8,SPACA4,SPAG16,SPAG17,SPAG5,SPOCK3,SPTBN4,SSTR4,ST18,STIL,STK31,STK32B,STK33,SYN2,SYNGR3,SYT3,TAC3,TACSTD2,TAFA1,TECTA,TERC,TEX11,TEX15,TFAP2A,TICRR,TM4SF5,TM7SF2,TMC5,TMEM236,TMEM63C,TMEM72,TMIGD1,TOP2A,TPBG,TPX2,TRANK1,TRIM10,TRIM31,TRIM63,TROAP,TRPM1,TRPM5,TRPM8,TRPV3,TSNAXIP1,TTLL9,TUB,TYMP,UBASH3A,UBD,UBE2C,UBE2T,UGT1A3,UGT1A4,UGT3A1,ULK4,UPK1A,UPP2,WDR64,WIF1,WNT2B,XIST,ZFYVE28,ZNF616,ZNF804B,ZNF93
Liver Failure	ADRA1D,ADRA2C,ADRB3,IL23R,OPRL1,SSTR4,TOP2A
Liver Damage	ADORA3,ALDH3A1,CCL2,CHRNA10,CTLA4,GABRB2,HBA1/HBA2,HLA-A, IL23R,LTA,MMP9,MPL,OPRL1,PTGER1, PTGS2,RGN,S100A4,SAA1,TACSTD2,TNFSF4,TSLP
Liver Inflammation	ADORA3,CCL2,CCL3L3,CHRNA10,CTLA4,CXCL2,CYP3A5,FOXP3,GABRB2,Havcr1,HBA1/HBA2,HLA-A,IL23R,LTA, MMP9,MPL,OPRL1,S100A4,SLC5A2,XCL1
